# Salaried and voluntary community health workers: exploring how incentives and expectation gaps influence motivation

**DOI:** 10.1186/s12960-019-0387-z

**Published:** 2019-07-19

**Authors:** Hermen Ormel, Maryse Kok, Sumit Kane, Rukhsana Ahmed, Kingsley Chikaphupha, Sabina Faiz Rashid, Daniel Gemechu, Lilian Otiso, Mohsin Sidat, Sally Theobald, Miriam Taegtmeyer, Korrie de Koning

**Affiliations:** 1 Royal Tropical Institute, KIT Health, P.O. Box 95001, 1090 HA Amsterdam, The Netherlands; 2 Eijkman Institute for Molecular Biology, Jalan Diponegoro 69, Jakarta, 10430 Indonesia; 3 Research for Equity and Community Health (REACH) Trust, P.O. Box 1597, Lilongwe, Malawi; 4 BRAC James P. Grant School of Public Health, BRAC University, Mohakhali, Dhaka Bangladesh; 5 HHA-YAM, P.O. Box 303, Hawassa, Ethiopia; 6 LVCT Health, Research and Strategic Information Department, P.O. Box 19835-00202, Nairobi, Kenya; 7 Department of Community Health, University Eduardo Mondlane, P.O. Box 257, Maputo, Mozambique; 8 Department of International Public Health, Liverpool School of Tropical Medicine, Pembroke Place, Liverpool, L3 5QA UK

**Keywords:** Community health workers, Motivation, Remuneration, Incentives, Low- and middle-income countries

## Abstract

**Background:**

The recent publication of the WHO guideline on support to optimise community health worker (CHW) programmes illustrates the renewed attention for the need to strengthen the performance of CHWs. Performance partly depends on motivation, which in turn is influenced by incentives. This paper aims to critically analyse the use of incentives and their link with improving CHW motivation.

**Methods:**

We undertook a comparative analysis on the linkages between incentives and motivation based on existing datasets of qualitative studies in six countries. These studies had used a conceptual framework on factors influencing CHW performance, where motivational factors were defined as financial, material, non-material and intrinsic and had undertaken semi-structured interviews and focus group discussions with CHWs, supervisors, health managers and selected community members.

**Results:**

We found that (a mix of) incentives influence motivation in a similar and sometimes different way across contexts. The mode of CHW engagement (employed vs. volunteering) influenced how various forms of incentives affect each other as well as motivation. Motivation was negatively influenced by incentive-related “expectation gaps”, including lower than expected financial incentives, later than expected payments, fewer than expected material incentives and job enablers, and unequally distributed incentives across groups of CHWs. Furthermore, we found that incentives could cause friction for the interface role of CHWs between communities and the health sector.

**Conclusions:**

Whether CHWs are employed or engaged as volunteers has implications for the way incentives influence motivation. Intrinsic motivational factors are important to and experienced by both types of CHWs, yet for many salaried CHWs, they do not compensate for the demotivation derived from the perceived low level of financial reward. Overall, introducing and/or sustaining a form of financial incentive seems key towards strengthening CHW motivation. Adequate expectation management regarding financial and material incentives is essential to prevent frustration about expectation gaps or “broken promises”, which negatively affect motivation. Consistently receiving the type and amount of incentives promised appears as important to sustain motivation as raising the absolute level of incentives.

## Background

Pursuit of ambitious global health initiatives, including those related to the 2015 sustainable development goals, led to renewed attention for health systems’ weaknesses [1]. Key challenges include public sector human resource shortages and their effects on access to services [2–6]. In response, health systems have (again) turned to engaging community health workers (CHWs) to reach out to underserved communities. The evidence of the potential contribution of CHWs to improving the health of populations is well documented [7–10], and there is renewed attention for the potential of strengthening the performance of CHWs; this is also evidenced by the recent publication of the World Health Organization (WHO) guideline on support to optimise CHW programmes [11].

### Community health workers

CHWs, who often are from low socio-economic backgrounds, have been defined as “any health worker carrying out functions related to health care delivery; trained in some way in the context of the intervention, and having no formal professional or paraprofessional certificate or degree in tertiary education” [8]. In addition, the WHO has suggested CHWs should be supported by the health system even when they are not a formal cadre [12]. They are the first point of contact between health service clients and providers, linking communities to the health system [13, 14]. CHWs deliver a wide range of promotive, preventive and partly curative services, including maternal and child health, HIV care and treatment of malaria [15, 16]. They are also in the unique position of being able to bring insights about community health to higher-level health workers [17]. CHWs mostly work in public and non-governmental settings in low- and middle-income countries (LMICs).

### CHW motivation, performance and incentives

**Fig. 1:**
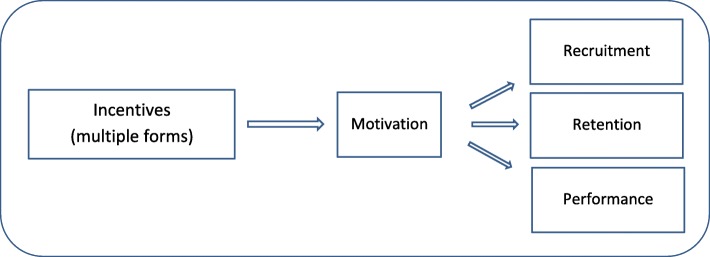
Model of the relationship between incentive types, motivation and CHW work behaviour (Daniels et al. 2014 [20])

The ability of CHWs to deliver effective services depends on many different contextual, health system and intervention design factors [14, 16, 18, 19]. CHW recruitment, retention and performance are enhanced by, or may even depend on, CHW motivation [20], defined as “an individual’s degree of willingness to exert and maintain an effort towards organizational goals” [21]. Motivation in turn is influenced by incentives, which can range from community appreciation to uniforms, volunteer allowances and remuneration, among others [7, 12, 17, 22, 23]. The diagram in Fig. 1 depicts this relationship [20].

There has been much debate over the type, scope and combination of incentives that would improve CHW motivation. We define incentives as “the intrinsic and extrinsic factors aimed at positively influencing CHW motivation” [20, 24].

Extrinsic factors can take the form of financial, material or non-material incentives. Financial incentives range from fixed salaries for those formally employed and allowances for volunteers, to performance-based incentives. Material incentives may include health insurance, clothing or tools of the trade such as boots and backpacks. Non-material incentives include community recognition, preferential treatment and developing new skills. Intrinsic factors relate for example to witnessing positive change and personal growth [12, 24, 25]. Bicycles and other forms of transport, regular supplies, training opportunities and supervision are sometimes also perceived as incentives. We prefer to call these “job enablers”, as essentially they constitute the basic resources and enabling environment the health system should make available for CHWs to perform well [12, 20, 24].

Some form of incentives is essential for CHW motivation and programme effectiveness and is expected by CHWs and offered by most organisations [12, 13, 24–27]. Some go a step further and argue that paying CHWs is needed to recognise the importance of this role as part of the health system, ensure fairness in terms of “payment for meaningful work” and substantial time investments and in order to provide much needed income in often impoverished settings [28, 29]. The CHW role is often performed besides multiple (productive and reproductive) responsibilities of women, and some men, in rural areas [17, 30–33]. The new WHO guideline on CHW programme support also recommends CHWs should receive a financial package [11]. Yet others argue that offering financial incentives is not always an effective or desirable strategy, as this may undermine the volunteering spirit or make clients query CHWs’ motives [27, 34–37].

This paper aims to take a critical look at the use of incentives and their link with improving CHW motivation, and how the salaried vs. volunteer status of CHWs and gaps in CHW expectations influence this link.

## Methods

This study is a comparative analysis of qualitative studies carried out in Bangladesh, Ethiopia, Kenya, Indonesia, Malawi and Mozambique. These countries were part of the REACHOUT research programme (2013-2018) focusing on factors influencing CHW performance [38]. The countries have well-established CHW programmes but considerable variation in CHW typology.

In 2013, REACHOUT programme undertook an initial international literature review [39] and developed a conceptual framework modelling the factors influencing health worker performance, with a special focus on CHWs [19, 25]. Using this unifying framework approach [40], the six countries derived topic guides for semi-structured interviews (SSIs) and focus group discussions (FGDs) with purposefully sampled CHWs, their supervisors, health managers and selected community members. SSIs were used to enable individual discussion (and took place in homes and offices) and allowed for sensitive areas to be probed, avoiding possible issues of power which can shape group discussion. FGDs used group interaction to generate findings to help understand community and organisational norms, common health issues and the need for access and use of healthcare services [41]. Sampling of study participants was based on their involvement in CHW programmes; variation was achieved based on demographic characteristics of participants and geographical characteristics of study sites. All six country studies explored participants’ perspectives on factors influencing CHW motivation, addressing incentives in terms of expectations and actual (perceived) incentives. Altogether, 250 interviews and 65 FGDs were undertaken as part of the six country qualitative context analyses, referred to as the REACHOUT country studies [42–47]. Details of data collection and respondents per country are presented in Table 1; these as well as details regarding study design (participant selection, setting, data collection), data analysis and reporting can be found in detail in the referred six REACHOUT country study reports.

**Table 1:** Interviews and focus group discussions conducted per country, by informant type (2013–2014)

	Ethiopia	Kenya	Malawi	Mozambique	Bangladesh	Indonesia
CHWs						
FGDs	HEWs—6	CHWs—6	HSAs—3			Village midwives and village nurses—3 *Kader*—8
SSIs	HEWs—12		HSAs—8	APEs—18	Formal CTCPs—8 Informal CTCPs—16	Village midwife and village nurses—44
CHW supervisors, managers, key informants						
SSIs	*Kebele* administrator—3 Health centre in charge—3 Delivery case team leaders—3 HEP coordinators—3 Regional HEP coordinator—1 Zonal HEP coordinator—1	CHEWs—16 SCHMT members—3 Facility in charges—4 National level policy makers—4	District level staff—13 Health centre in charges—2 NGO staff—9	Health facility supervisors—3 District supervisors—2	Paramedic—2 Clinic manager—2 Counsellor—2 Nurse—1 Program officer—1	Head of PHC or *Puskesmas*—4 Midwife coordinator—2 Head of district MCH section—2
Community members						
FGDs	Women—6 Men—2	Community members—4	Women—7 Volunteers—6	Mothers—8	Married women—8 Married men—4	Men—2
SSIs	Mothers—12 TBAs—6	Community members—10	Mothers—1 TBAs—6 Traditional leaders—3 Volunteers—2	Community leaders—6		Mothers—39 TBAs—8 Head of village and head of PKK—17

APE *Agentes polivalentes elementares* (elementary multipurpose agents), CHEW community health extension worker, CHW community health worker, *CTCP* close-to-community provider, *FGD* focus group discussion, *FWA* family welfare assistant, *HEP* health extension programme, *HEW* health extension worker, *HSA* health surveillance assistant, *Kader* volunteer CHW, *MCH* Maternal and child health, *PHC* primary health care, *PKK Pembinaan Kesejahteraan Keluarga* (refers to the “family welfare movement”—an Indonesian women’s organisation), *SSI* semi-structured interview, *NGO* non-governmental organisation, *Puskesmas* sub-district community health centre, *SCHMT* sub-county health management team *TBA* traditional birth attendant

For the current study, we performed a comparative analysis of the existing datasets of the six country studies, analysing them from various perspectives. We started off with a cross-country review of the six country study reports. To further deepen the themes that emerged from the initial analysis, we then performed secondary data analysis by reviewing existing datasets in Nvivo 10, for codes related to incentives (various forms of incentives and disincentives, motivation, job satisfaction, training, career advancement). Main themes were identified, narratives developed and contextual comparative analyses conducted to explore how the realities of CHWs’ role within different countries shaped their experiences and expectations (Table 2).

**Table 2:** CHW typology in the six study countries by type of organisation and mode of engagement

Mode of engagement	Country: CHW type	Incentives		
		Financial incentives, *as agreed*		Material incentives, *as agreed*
		Per month (2014 USD equivalent), *as agreed*	% of starting salary enrolled nurse, per month (2014 USD equivalent)	
Contracted staff, with salary	Government (MoH)			
	Bangladesh: FWAs	USD98	48% (USD206)	
	Ethiopia: HEWs	USD46	74% (USD62)	Sometimes: airtime
	Malawi: HSAs	USD100	13% (USD778)	Sometimes: bicycles, t-shirts, airtime, uniform
	NGO			
	Bangladesh: *Shasthya kormis (SK)*	USD39	NA	
Volunteers, with some form of financial incentive *Note: time inputs vary across countries/cadres*	Government (MoH)			
	Indonesia: *kader*	Allowance: USD5 (but variable)	3% (USD200)	Uniform/t-shirt
	Kenya: CHWs	Allowance: USD23	10% (USD226)	Sometimes: bicycles
	Mozambique: APEs	Allowance: USD40	33% (USD120)	Uniform Sometimes: bicycles, airtime
	NGO Bangladesh: *Shasthya shebikas (SS)*	Performance-based incentives: varies; selling commodities: varies (average around USD3 per month)	NA	

APE *Agentes polivalentes elementares* (elementary multipurpose agents), *CHW* community health worker, *FWA* family welfare assistant, *HEW* health extension worker, *HSA* health surveillance assistant, *kader* volunteer CHW, *MoH* Ministry of Health, *NA* not available, *NGO* non-governmental organisation, *SK* health workers, *SS* health volunteers, *USD* United States dollars

## Results

Across all six study settings, incentives were found to be an important factor influencing motivation of CHWs and are shaped by three key emerging themes: the mode of CHW engagement (employed vs. volunteering) influences how various forms of incentives affect each other and motivation, incentive-related “expectation gaps” negatively influence motivation, incentives can cause friction for the interface role of CHWs between communities and the health sector.

### The mode of CHW engagement influences how various forms of incentives affect each other and motivation

*Financial incentives* were important in all study countries, whether they take the form of salaries or allowances. The appreciation of the salaries CHWs received in Bangladesh, Ethiopia and Malawi as contracted staff demonstrated this. CHWs in Bangladesh, Indonesia, Kenya and Mozambique also welcomed the small monthly volunteering allowance they received.

“Since I am paid my salary, I am committed to serve the community. I am happy when I work hard and take my salary.” (Salaried HEW, SSI-Ethiopia)

While salaries were seen as “part of the package” by employed CHWs, voluntary CHWs did not always take their allowance for granted. This was among others the case in Indonesia, where CHWs (*kader*) said they did not always receive an allowance as per policy.

*Material incentives* were seen as very important factors affecting CHW motivation. While CHWs sometimes received food or other goods from the communities they served, receipt (or not) of this type of incentive was linked to the organisation they worked with. This often took the form of uniforms and backpacks, as well as job enablers, such as bicycles and standard kits with supplies and drugs.

“So those [CHWs] with bicycles, you find they are active. Those who do not have, you find that they are challenged. So I would think if each had a bicycle, it would become a lot better.” (Supervisor of voluntary CHWs, SSI-Kenya)

Such material incentives and job enablers not only supported them in their work, but also served to reinforce the standing of community health programmes, both for CHWs and the communities they served.

Material incentives and job enablers were important to employed as well as voluntary CHWs, albeit reasons for this partially differed. For employed CHWs who are sure of receiving a salary, material incentives seemed to be less important; however, the absence of material incentives constituted a disincentive, as we will elaborate below. At the same time, many employed CHWs felt their salary is (too) low, which may make material incentives more important. This also applied to voluntary CHWs, as allowances were usually small. When the intervention design failed to include material incentives, CHW motivation was hindered and programme effectiveness undermined, regardless of the presence of financial incentives.

“I have seen something that makes [CHWs] not to work well. They don’t have transport. They don’t have enough support to motivate them. Because the work they do is voluntary.” (Supervisor of voluntary CHWs, SSI-Kenya)

CHW respondents in all study countries (regardless of whether they were employed or volunteers) mentioned that they appreciated *non-material incentives*; many indicated that these helped sustain motivation and made them feel that communities value and appreciate them, recognising the positive contribution of their work.

“A thing that pushes me to work hard is the satisfaction of community by my services. (…) When I see the output of my work, I am very happy.” (Salaried HEW, FGD-Ethiopia)

Other examples of non-material incentives were feelings of pride when the community “responds” to health education work and being happy with knowledge and skills obtained, encouragement from supervisors and preferential treatment at the health facility.

Sometimes CHWs received less than expected community appreciation; this was a de-motivator that seemed to affect voluntary CHWs more than those salaried as it hurts the core of the volunteering spirit:

“Those things that don’t do me good is that you can get to a certain household and he or she doesn’t want to respond. Imagine coming to help, am not paid, have left my own work and then he or she sees me as no one.” (Voluntary CHW, FGD-Kenya)

In Bangladesh, most CHWs (both employed and volunteers) reported that being appreciated, recognised and valued by the communities they serve was more important than the financial income they generated through providing these services.

Examples of *intrinsic motivational factors* were also found in all study countries. Most CHWs desired to assist fellow community members and felt they are useful to their communities; these were major motivational factors, both for salaried and voluntary CHWs. Other intrinsic factors mentioned included love for voluntary work and the perception that as a CHW one is the key link between the health facility and the community and it being “God’s will”. Some CHWs insisted that monetary incentives were less important than intrinsic motivation; this was more common among voluntary than salaried CHWs.

“The *kader* have to have the principle that this work is voluntarily done, without asking for salary; if the salary is available, just be grateful.” (Voluntary *kader*, SSI-Indonesia)

In addition, several Kenyan respondents observed that extrinsic incentives and job enablers can sustain intrinsic volunteerism motivation and that lack of such incentives can lead to demotivation.

“This service of a CHW is a calling (…). I have a family, my family needs to eat, my children need to study (…). I do not have a salary, many CHWs live in sad places (…). It is my prayer that we get paid at the end of the month and this will motivate us to work.” (Voluntary CHW, FGD-Kenya)

On the other hand, others felt that the introduction of financial incentives could weaken intrinsic motivation of volunteers, as was observed in Indonesia:

“I think they were more active before they got incentives. They worked voluntarily and not for money.” (Health manager on voluntary *kader*, SSI-Indonesia)

### Incentive-related “expectation gaps” negatively influence motivation

In each country setting, employed and voluntary CHWs expressed that when their expectations regarding incentives were not met this negatively affected their motivation. Expectation gaps took various forms: lower than expected financial incentives, later than expected payments, fewer than expected material incentives and job enablers and unequally distributed incentives across groups of CHWs.

*First*, employed CHWs often felt their salaries were too low in comparison with their workload.

“Our salary is very unfair, with our responsibility and the services we are giving for our society.” (Salaried HEWs, FGD-Ethiopia)

Many respondents observed the same for voluntary CHWs, who felt that their monetary compensation was (far) below expectations. The importance of financial rewards and disappointment about the amount may also be linked to the poor socio-economic backgrounds the CHWs in our study sites hailed from and whether this work was their primary source of income.

*Second*, whenever financial incentives of CHWs were expected to be paid but where bureaucratic or other problems caused late disbursements, CHWs became demotivated. Many CHWs reported receiving less than expected or no financial incentives at all; some felt forced to use their own resources to be able to do their job.

“This subsidy is just not enough for anything, but they promised us and should at least give us the little at the end of the month, and they give just nothing. (…) I have to support my family.” (Voluntary APE – *Agente polivalente elementar* (elementary multipurpose agent), SSI-Mozambique)

*Third*, in most of the countries, CHWs observed gaps between promised and received material incentives, such as uniforms and (job enabling) standard kits. Many CHWs also expected other material incentives.

“It requires an HSA to be given transport, identity card, uniform and gumboots as our friends were given but we have never been given.” (Salaried HSA, SSI-Malawi)

In all countries, CHWs observed important gaps between expected and actual job enablers, which acted as disincentives for CHWs. CHWs found supportive supervision irregular or not supportive and administration-oriented, in a majority of the countries.

“[What does not please me is] being belittled by my seniors, who see us as nothing and that we are doing this work because we have nothing better to do.” (Voluntary CHW, FGD-Kenya)

CHWs’ expectations regarding professional development were often not met, as observed by respondents in Ethiopia, Malawi and Mozambique. Lack of training opportunities was seen as contributing to the lack of career opportunities in the same countries, to CHWs’ frustration. In most countries, CHWs’ expectations regarding transport for home visits and sometimes also referrals were not met.

“Everybody is developing in his career; we health extension workers remain where we are. (…) These things are pushing us backward not to work hard.” (Salaried HEW, FGD-Ethiopia)

“We were promised motorbikes and bicycles and yet they have not been fulfilled. False promises are really frustrating.” (Voluntary CHW, FGD-Kenya)

Also, CHWs in Ethiopia, Indonesia, Malawi and Mozambique noted the “inadequate” provision of equipment and regular supplies to perform their duties, leading to frustration and demotivation.

*Fourth*, as there were no standardised incentive structures for CHWs, incentive-related “competition” between programmes employing CHWs affected motivation negatively.

“Those who are in government facilities get allowances for outreach clinics, while here [at CHAM - Christian Health Association of Malawi] we work for nothing if we go for outreach clinics and in terms of transport. In government facilities they are considered with a motorcycle, but in CHAM not.” (Salaried HSA, FGD-Malawi)

Such inequity disturbed the functioning of CHWs, by subverting the incentive structures, thus challenging underlying motivational processes. Another inequity surfaced when CHWs perceived the way material incentives were distributed as unfair, due to favouritism and lack of transparency, as found in Ethiopia, Kenya and Malawi. Ultimately, this undermined motivation and the functioning of community health interventions.

“You will find that [the supervisor] will only invite the few secretly [to receive some compensation] and not all the CHWs. (…) This is something that makes us demotivated and even think of withdrawing because he is not transparent.” (Voluntary CHW, FGD-Kenya)

### Incentives cause friction for the interface role of CHWs between communities and the health sector

CHWs are playing a key facilitation and referral role between communities and the health sector. Incentives were sometimes observed to establish and strengthen relationships and accountability, especially between CHWs and their supervisors. This applied to both salaried and voluntary CHWs.

Conversely, lack of promised payments sometimes led to mistrust towards those in the health sector, while demotivation sometimes led to reduced community trust in CHWs, thus undermining the effectiveness of community health programmes.

“This job is very hard to do (…). This payment we are supposed to get, the government is the one that is not trustworthy. They should give some money for us to be paid every month.” (Voluntary CHW, FGD-Kenya)

A Kenyan key informant observed that absence of a government salary means a (volunteer) CHW cannot be held accountable, in contrast with (salaried) community health extension workers (CHEWs).

“Because CHEWs are paid by the government you can hold them to account, rather than the volunteer [CHW] who can leave an important job half way and you cannot hold him/her accountable because they were volunteering.” (Policy maker, SSI-Kenya)

The effect of payment on CHW accountability towards communities remains to be seen; there was some evidence that (perceptions about) payment of CHWs could result in community mistrust.

“The community (…) will regard us as responsible and meaningful people to the community. So even if they say we get paid we just keep silent (…). What I don’t like is them mocking me, you can get to some places where they say we are paid and also they are speaking in mockery tones.” (Voluntary CHW, FGD-Kenya)

## Discussion

Our study findings from six different contexts contribute to debates on how various types of incentives, alongside job enablers, influence not only motivation but also each other; and what the role is of CHW engagement (salaried vs. voluntary) and the price of “false promises”.

Our findings demonstrate that both financial and non-financial incentives, independently and together, improve CHW motivation [12, 14, 32]. Monetary compensation is highly valued and directly motivates CHWs to perform better and allows them to dedicate themselves to their jobs, whether in a salaried or volunteering position. Non-financial incentives (material and non-material) can, with or without accompanying financial incentives, also contribute to motivation. However, financial incentives alone seem insufficient to maintain motivation and need to be complemented by other types of incentives and job enablers like training and supplies, among others [11, 24, 25, 32, 48]. Both salaried and allowance-receiving voluntary CHWs in all study contexts showed that intrinsic motivational factors were also important to them, confirming findings elsewhere [24, 27, 49].

Discussion around a volunteering approach vs. providing salaries to CHWs is ongoing [13, 28, 34, 49]. Volunteers are, generally, unlikely to continue to serve without a clear prospect of receiving salaries, especially as expectations in the form of tasks and workload expand [30–32]. Lack of salaries contributes to attrition within voluntary CHW programmes and makes regular recruitment and training of new volunteers necessary [13, 30, 48, 50].

The mode of engagement of CHWs affects the way incentives influence each other, as well as motivation. Employed, salaried CHWs often seem demotivated due to the perceived low level of monetary reward as compared to workload and living costs [49]. Connectedness to communities serving as intrinsic motivation is important to many salaried CHWs but cannot easily compensate for the perceived low financial reward. The reverse seems true for volunteers: the strong intrinsic motivators identified by many voluntary CHWs prevent them to become dispirited about their modest allowances. Some explicitly indicate such financial incentives are “nice” but not as important as the intrinsic factor driving their volunteering spirit. In contrast, we also found some evidence that extrinsic incentives and job enablers can sustain intrinsic volunteerism motivation and that lack of such incentives can lead to demotivation. At the same time, one statement of a manager in Indonesia implied that the introduction of financial incentives for volunteers who previously did not receive an allowance weakened intrinsic motivation. This potential negative effect of financial incentives has been highlighted by other authors [24, 34, 48, 49, 51, 52].

While our findings indicate that the way incentives influence motivation depends at least in part on individual behaviour, this is in turn influenced by context. Strachan et al. [53] have argued that CHW motivation may increase relevant to the degree CHWs identify with group membership and share a collective social identity. Their suggestion to use the Social Identity Approach theory to help understand CHW motivation and design related interventions is worthwhile to explore further.

Incentives do not automatically lead to improved motivation as expected. Authors over time have consistently referred to enabling factors that should be present to ensure this [20, 24]. Indeed, our findings suggest that several job enablers need to be in place but often were not, including (i) minimum resources to perform well, such as a functioning supply chain to enable implementation of core tasks (e.g. for test kits and drugs) and some form of transport where distances are unfeasibly long, for CHWs to do their usual work and for CHW clients to be referred; (ii) regular, supportive oriented (as opposed to fault-finding or purely administrative-oriented) supervision; and (iii) continuous education and professional development opportunities. Other studies confirm this, also indicating that where any of these job enablers is felt missing or much weaker than anticipated, they are a barrier or disincentive to CHW performance [13, 17, 33, 49, 54–56]. The WHO guideline on support to CHW programmes makes explicit recommendations towards ensuring each of these three enabling areas [11].

**Fig. 2:**
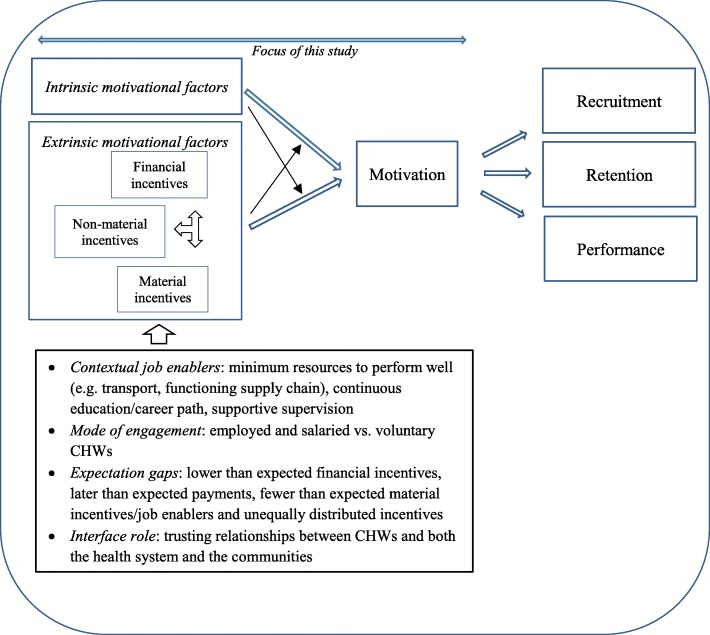
Revised model: relationships between motivational factors, motivation and CHW work behaviour. CHW = community health worker

In line with the above, Fig. 2 presents our revised, more detailed model to visualise the way incentives interact with each other and with motivation, and the way job enablers and CHW engagement influence incentives and motivation.

The findings confirm that both salaried and voluntary CHWs mostly feel that the monetary compensation they receive is not commensurate with their efforts; this is likely to be an important issue in poor socio-economic contexts. Lower-than-expected incentives and not receiving promised (financial or material) incentives lead to demotivation and are a barrier to performance [37, 57–59]. It is noteworthy that many CHWs appear to feel more strongly about “broken promises” regarding incentives and job enablers, than about the absolute level of incentives received.

Incentive packages therefore should be in line with CHW job demands, in terms of expectations regarding type and number of tasks, training, as well as expected time investment [11, 33, 58]. As important is that such commitments then also are honoured.

Health systems are increasingly expected to be accountable to the communities they serve. Authors have argued that remuneration may lead to CHWs feeling more accountable towards the health sector than to their communities [60–62]. Reversely, if CHWs engaged by the health system are not paid or otherwise formally embedded, there is no valid mechanism for the system to hold them accountable for their performance [17, 63].

Our findings show that (especially financial but also material) incentives indeed may serve to establish and strengthen relationships of accountability between CHWs and their supervisors within the health system. If financial incentives take the form of a salary tied to a position as government staff, CHWs are well-integrated into the health system and this can lead to increased effectiveness [37]. Conversely, not receiving promised monetary incentives (whether a salary or allowance) in time or not at all creates mistrust towards the health system [14, 48].

Financial incentives may also put in jeopardy the accountability relationship with the community. Mistrust between CHWs and the communities they serve is sometimes created when community members perceive allowances as “salary” and then interpret voluntary CHWs as formally being part of the health sector and make proportionate (demanding) claims on their support. Another study found that community members may sometimes think CHWs withhold money or earn money “behind their backs” [64].

The current paper is based on an analysis of existing datasets from a broader study on factors influencing the performance of CHWs in six country contexts. Although different types of incentives were part of data provided by study respondents, it is possible that more in-depth information about incentives could have added to the appreciation of factors influencing the way incentives operate in the countries studied. While each country study included the same categories of respondents, there were differences in types and gender of respondents between countries (Table 1), which could have influenced the findings. In addition, CHW programmes across the world have many diverse features and contexts, which pose limitations to the comparison and the generalisability of study findings. To support generalisability, we have tried to provide clear information about the programme features and contexts of the CHW programmes studied in the six countries, and also discussed where findings are common or differing across the six REACHOUT country contexts.

## Conclusions

Whether CHWs are employed or engaged as volunteers has implications for the way incentives influence motivation. While intrinsic motivational factors are important to and experienced by both types of CHWs, for many salaried CHWs, they may not suffice to compensate for the demotivation that often exists and is arising from the perceived low level of financial reward. In some contexts, voluntary CHWs may not take allowances for granted and continue to rely on intrinsic motivation to carry on with their work. Yet overall, introducing and/or sustaining a form of financial incentive seems key towards strengthening CHW motivation. Such remuneration should then be fair (commensurate to the job demands) and consistent (over time as well as across CHW groups with similar job demands).

There is some evidence that extrinsic incentives and job enablers can sustain intrinsic motivation for volunteerism. Adequate expectation management regarding financial and material incentives as well as job enablers is essential, to prevent frustration about expectation gaps or “broken promises” to negatively affect motivation. For many CHWs, consistently receiving the type and amount of incentives promised appears as important to sustain motivation as raising the absolute level of incentives.

## Data Availability

The datasets generated and analysed during the current study are available from the country study authors on reasonable request; contact details are available through the corresponding author.

## Abbreviations

APE: *Agentes polivalentes elementares* (elementary multipurpose agents, Mozambique)
CHAM: Christian Health Association of Malawi
CHEW: Community health extension worker (Kenya)
CHW: Community health worker
FGD: Focus group discussion
HSA: Health surveillance assistant (Malawi)
HEW: Health extension worker (Ethiopia)
LMICs: Low- and middle-income countries
NGO: Non-governmental organisation
SSI: Semi-structured interview
WHO: World Health Organization
